# Ni-Substitution-Induced Microstructural Evolution and Electromagnetic Loss Regulation in U-Type Hexaferrite Ceramics for Enhanced Microwave Absorption

**DOI:** 10.3390/ma19214617

**Published:** 2026-10-30

**Authors:** Gangli Feng, Yupeng Zhang, Ziyu Guo, Liangzhi Chen, Fuyuan Dong, Yan Zhao, Yanru Liu, Ning Li

**Affiliations:** 1 School of Materials Science and Engineering, North Minzu University, Yinchuan 750021, China; 2 International Scientific & Technological Cooperation Base of Industrial Waste Recycling and Advanced Materials, Yinchuan 750021, China; 3 Ningxia Key Laboratory of Powder Materials Intelligent Design and Manufacturing, Yinchuan 750021, China; 4 Ningxia Hui Autonomous Region Agricultural Comprehensive Development Office, Yinchuan 750021, China

**Keywords:** U-type hexaferrite ceramics, Ni substitution, solid-state reaction, dielectric properties, magnetic properties, microwave absorption

## Abstract

**Highlights:**

- Ni substitution tailors the electromagnetic response of U-type hexaferrites.
- The x = 0.2 sample achieves a broad 9.3 GHz absorption bandwidth.
- Microwave absorption is optimized through attenuation-matching balance.

**Abstract:**

U-type hexaferrites Ba_4_Co_2_Fe_36−x_Ni_x_O_60_ (x = 0.0–0.4) with nominal Ni substitution were prepared by a conventional solid-state reaction. X-ray diffraction analysis indicates that all samples are predominantly composed of the U-type phase. Scanning electron microscopy observations reveal that the samples exhibit faceted and polyhedral grains with polygonal and triangular outlines. With increasing Ni content, the magnetic properties are effectively regulated, with the saturation magnetization increasing from 12.8 emu·g^−1^ to 14.5 emu·g^−1^ and the coercivity exhibiting a non-monotonic variation. Meanwhile, the complex permittivity and permeability are systematically tuned, leading to improved electromagnetic attenuation capability and impedance matching. At a thickness of 6.0 mm, the x = 0.2 sample exhibits the broadest absorption bandwidth, defined as a reflection loss of 9.3 GHz (RL ≤ −5 dB), whereas the x = 0.4 sample achieves a minimum reflection loss (RL_min_) of −36.0 dB and an effective absorption bandwidth of 6.8 GHz (RL ≤ −5 dB). These results reveal that Ba_4_Co_2_Fe_36−x_Ni_x_O_60_ is a great potential absorber in electromagnetic (EM) wave absorption.

## 1. Introduction

High-frequency electronic devices and wireless communication are developing rapidly. As a result, electromagnetic pollution and interference are becoming more serious, affecting normal equipment operation and potentially harming human health. Thus, microwave-absorbing materials have again attracted considerable attention [1,2,3]. An ideal absorber should be thin, light, wide, and strong—meaning small thickness, low density, wide absorption bandwidth, and high absorption intensity [4,5]. Compared with conventional dielectrics, hexagonal ferrites offer clear advantages for high-frequency absorption because of their good chemical stability, strong magnetic response, and high Snoek limit [6,7,8].

Among the many hexagonal ferrites, U-type Ba_4_Co_2_Fe_36−x_Ni_x_O_60_ has attracted attention because of its tunable magnetic-moment distribution and magnetocrystalline anisotropy field. U-type hexaferrites possess a complex rhombohedral crystal structure with the R-3m space group. The crystal structure contains multiple nonequivalent crystallographic sites for magnetic cations, including octahedral, tetrahedral and trigonal bipyramidal coordination environments. The magnetic properties of U-type hexaferrites are strongly influenced by the distribution of Fe and transition-metal ions among these sites, which determines the magnetic sublattice configuration and exchange interactions [9,10,11]. Cation substitution is an effective strategy for tailoring the permeability, permittivity and magnetic response of hexagonal ferrites [12,13,14]. However, achieving high-performance microwave absorption in U-type hexaferrites remains challenging because strong electromagnetic attenuation does not necessarily guarantee efficient absorption. Excessive dielectric or magnetic loss may deteriorate impedance matching, whereas insufficient attenuation limits electromagnetic energy dissipation within the absorber. Therefore, rational regulation of magnetic response, dielectric polarization and impedance matching is essential for optimizing microwave absorption performance. Although cation substitution has been widely explored in hexagonal ferrites, the relationship between substitution-induced microstructural evolution, magnetic loss behavior and the resulting balance between attenuation capability and impedance matching in U-type hexaferrites remains insufficiently understood [15,16,17,18].

According to Shannon’s ionic radii, the ionic radius of octahedrally coordinated high-spin Ni^2+^ is 0.69 Å, while that of octahedrally coordinated high-spin Fe^3+^ is 0.645 Å [19]. The magnetic moment of Ni^2+^ is smaller than that of Fe^3+^. Owing to the differences in ionic radius and magnetic moment between Ni^2+^ and Fe^3+^, nominal Ni substitution may modify the local structural environment and magnetic interactions, thereby affecting the electromagnetic response. Moreover, cation substitution can adjust the balance between dielectric loss and magnetic loss, which is beneficial for impedance matching between the absorber and free space. Previous studies have shown that cation incorporation in U-type and related hexaferrites can tune electromagnetic parameters and microwave absorption performance through the balance between dielectric loss, magnetic loss and impedance matching. Nickel is a critical transition metal with growing demand from electric-vehicle and electronic applications. Developing Ni-substituted ferrite absorbers may contribute to the efficient utilization of Ni resources in microwave-absorbing applications. Therefore, it is necessary to further investigate the electromagnetic response and microwave attenuation behavior of Ni-substituted Ba-Co U-type hexaferrite ceramics.

In this study, Ba_4_Co_2_Fe_36−x_Ni_x_O_60_ (x = 0.0–0.4) U-type hexaferrite ceramics were prepared by a conventional solid-state reaction method. The phase evolution, microstructure, magnetic properties, complex permittivity and permeability, and microwave absorption performance were systematically investigated. Special attention was paid to the correlations among grain-size evolution, magnetic and dielectric loss, impedance matching and reflection loss. This work aims to provide an experimental basis for regulating the electromagnetic response of Ba-Co U-type hexaferrite ceramics for microwave absorption applications.

## 2. Materials and Methods

### 2.1. Materials and Sample Preparation

The solid-state reaction route was selected because of its simple process, low cost, and scalability for bulk ferrite production compared with wet-chemical methods such as sol–gel and co-precipitation. We purchased nickel oxide (NiO, 99.5% purity) from Shandong Xiya Chemical Co., Ltd. (Linyi, China), barium carbonate (BaCO_3_, 99.0%) from Shanghai Guangnuo Chemical Technology Co., Ltd. (Shanghai, China), iron oxide (Fe_2_O_3_) from Shanghai Shanpu Chemical Co., Ltd. (Shanghai, China), and cobalt oxide (Co_3_O_4_, 99.9% purity) from Shanghai McLean Biochemical Co., Ltd. (Shanghai, China). We used anhydrous ethanol (C_2_H_5_OH) as received.

We prepared Ba_4_Co_2_Fe_36−x_Ni_x_O_60_ (x = 0.0, 0.1, 0.2, 0.3, 0.4) powders using a solid-state reaction. We weighed BaCO_3_, Co_3_O_4_, Fe_2_O_3_, and NiO (precision 0.1 mg) according to the stoichiometric ratio. We ball-milled the mixture at 300 rpm for 3 h using anhydrous ethanol as the medium and zirconia milling balls in a planetary ball mill (YXQM-2L, MITR, Changsha, China) with a ball-to-powder ratio of 15:1. The slurry was then left to dry naturally at room temperature for 24 h. We pre-sintered the samples at 800 °C for 5 h in a muffle furnace with a heating rate of 5 °C/min. We ground the pre-sintered product and passed it through a 100-mesh sieve (approximately 150 μm). Polyvinyl alcohol (PVA) binder (5 wt% relative to the ferrite powder mass, added as an aqueous solution) was added to 6 g of the sieved powder. The mixture was ground again and pressed into circular green pellets with a diameter of 40 mm at 10 MPa for 5 min. After sintering, the samples had a circular-disk geometry with a diameter of approximately 35 mm and a thickness of approximately 1 mm. The green pellets were first heated to 600 °C at a heating rate of 5 °C·min^−1^ and held for 2 h to remove the binder. Subsequently, the pellets were sintered at 1100 °C for 2 h using the same heating rate and then furnace-cooled to room temperature; no programmed cooling rate was applied.

### 2.2. Characterization Methods

Phase structures were characterized by powder X-ray diffraction (XRD, SmartLab SE, Rigaku Corporation, Tokyo, Japan) using Cu Kα radiation (λ = 0.15406 nm) operated at 40 kV and 50 mA. The diffraction data were collected over the 2θ range of 5–90° with a scanning rate of 5°·min^−1^. Surface morphologies and grain-size distributions were observed using scanning electron microscopy (SEM, Sigma 360, Carl Zeiss Microscopy GmbH, Jena, Germany). Magnetic hysteresis loops were measured at room temperature using a vibrating sample magnetometer (VSM, Model 7404, Lake Shore Cryotronics, Westerville, OH, USA). The complex permittivity and permeability were measured in the frequency range of 2–18 GHz using a vector network analyzer (VNA, E5071C, Agilent Technologies, Santa Clara, CA, USA) and the coaxial-line method. The ferrite powders were mixed with paraffin at a mass ratio of 80:20, corresponding to 80 wt% ferrite and 20 wt% paraffin, and then formed into annular specimens for coaxial-line measurements. Because the density of the ground ferrite powder was not independently measured, the corresponding filler volume fraction was not determined. The annular specimens had an outer diameter of 7.00 mm, an inner diameter of 3.04 mm, and a thickness of approximately 2.00 mm. The vector network analyzer was calibrated using the short-open-load-through (SOLT) procedure. The complex permittivity and permeability were extracted from the measured scattering parameters using the Nicolson–Ross–Weir (NRW) algorithm. Surface elemental compositions and chemical states were further characterized by X-ray photoelectron spectroscopy (XPS, K-Alpha, Thermo Fisher Scientific, East Grinstead, UK). The binding energies were calibrated using the adventitious C 1s peak at 284.8 eV. The XPS spectra were processed and analyzed using Thermo Avantage software (version 5.9931, Thermo Fisher Scientific), including background subtraction, peak fitting and chemical-state analysis. For the representative x = 0.4 sample, the high-resolution Ni 2p spectrum was analyzed using a constrained fitting procedure with Shirley background subtraction and literature-guided component positions. Considering the weak Ni signal and the complex multiplet splitting and satellite structures of Ni 2p spectra, the fitting results were mainly used for qualitative identification of the Ni oxidation state rather than quantitative determination of the Ni^2+^/Ni^3+^ ratio.

## 3. Results

### 3.1. Phase Structure

**Figure 1:**
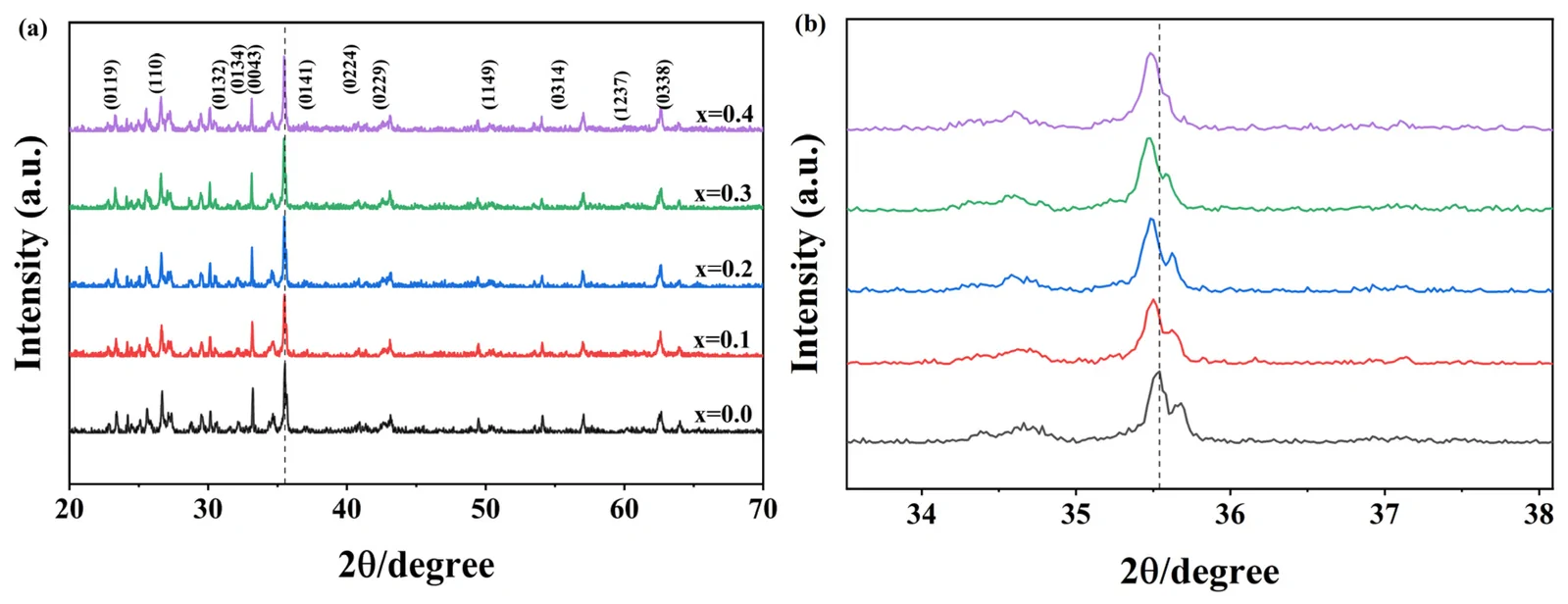
XRD patterns of Ba_4_Co_2_Fe_36−x_Ni_x_O_60_ ceramics with different Ni contents (x = 0.0–0.4): (**a**) XRD patterns over 2θ = 20–70° and (**b**) enlarged diffraction region. The diffraction peaks were indexed according to the reported U-type hexaferrite structure.

Powder X-ray diffraction (XRD) was used to characterize the phase composition of all Ba_4_Co_2_Fe_36−x_Ni_x_O_60_ (x = 0.0, 0.1, 0.2, 0.3, 0.4) samples. All measurements were collected using Cu Kα radiation (λ = 0.15406 nm). Figure 1a shows the XRD patterns of Ba_4_Co_2_Fe_36−x_Ni_x_O_60_ ceramics with different Ni contents. The complex crystal structure and large unit cell of U-type Ba–Co ferrites may also result in considerable peak overlap in laboratory XRD patterns. Therefore, the XRD results are used primarily to identify the main phase and assess qualitative phase evolution rather than to establish quantitative phase purity or specific cation-site occupancy. The diffraction peaks were indexed with the Co_2_U structure, and the assigned (hkl) planes are indicated in Figure 1. The obvious diffraction peaks at (0119), (110), (0132), (0134),(0043), (0141), (0224), (0229), (1149), (0314), (1237) and (0338) are consistent with those reported for U-type Ba–Co hexaferrites [20,21,22]. These results indicate that the main phase of all samples is U-type. The enlarged diffraction pattern in Figure 1b shows a slight peak shift with increasing nominal Ni content, suggesting a local lattice adjustment associated with Ni incorporation.

### 3.2. Microscopic Morphology

**Figure 2:**
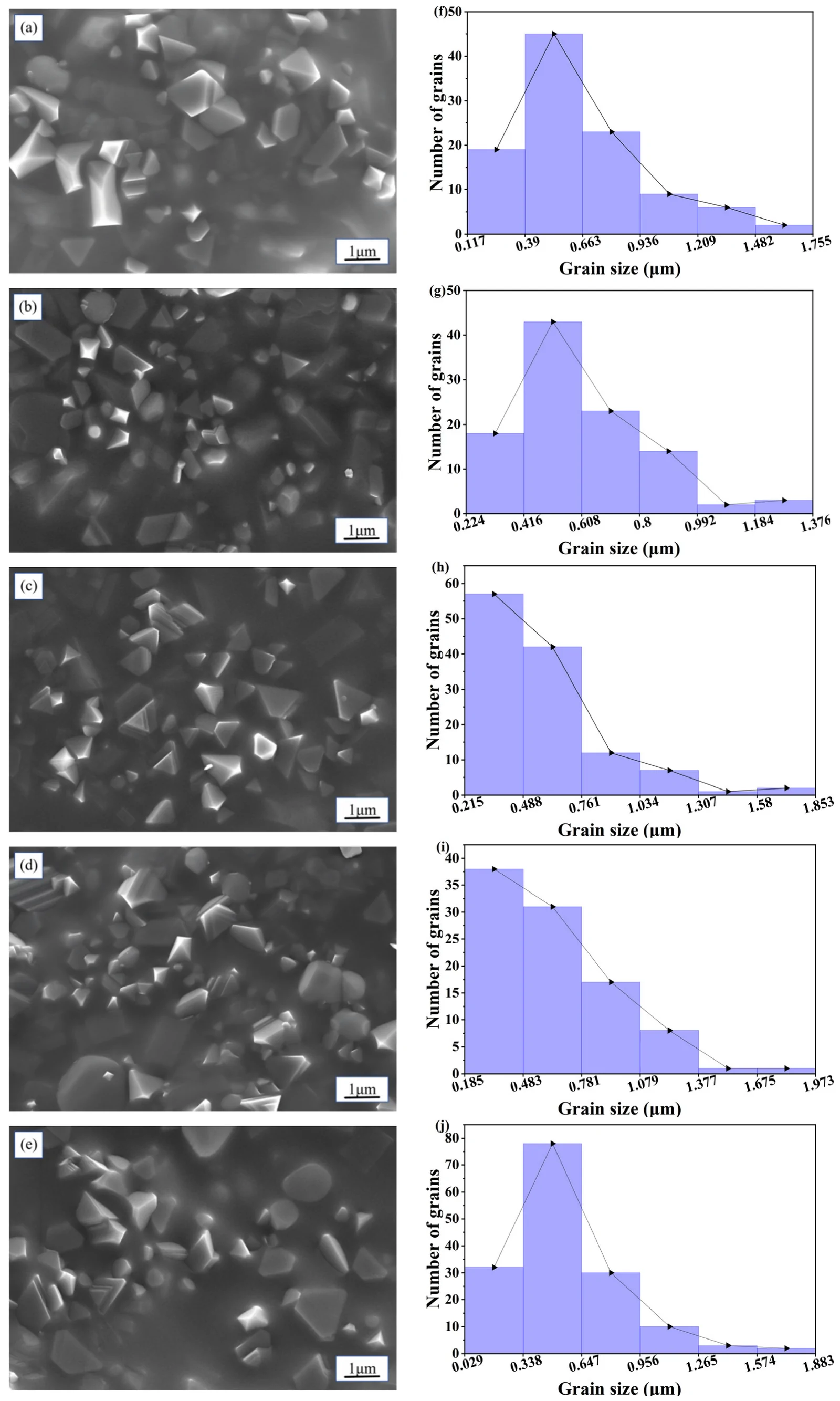
SEM images and corresponding grain-size distributions of Ba_4_Co_2_Fe_36−x_Ni_x_O_60_ ceramics with different Ni contents: (**a**–**e**) SEM images and (**f**–**j**) grain-size distributions, where (**a**,**f**), (**b**,**g**), (**c**,**h**), (**d**,**i**), and (**e**,**j**) correspond to x = 0.0, 0.1, 0.2, 0.3, and 0.4, respectively.

Figure 2 displays the SEM images of the samples with different nominal Ni contents (x = 0.0, 0.1, 0.2, 0.3, and 0.4). The SEM images predominantly show faceted and polyhedral grains with polygonal and triangular outlines, rather than well-defined hexagonal platelets. Because subtle differences in grain size are difficult to distinguish visually from individual SEM micrographs, quantitative statistical analysis was performed to characterize the grain-size evolution more objectively.

The mean grain sizes and standard deviations obtained from the analyzed grains are summarized in Table 1. The average grain sizes were 0.659 ± 0.324 μm for x = 0.0, 0.577 ± 0.294 μm for x = 0.1, 0.606 ± 0.231 μm for x = 0.2, 0.570 ± 0.290 μm for x = 0.3, and 0.647 ± 0.314 μm for x = 0.4. These values show a non-monotonic variation with increasing nominal Ni content. However, the differences among the compositions are comparable to the corresponding standard deviations. Statistical significance was not evaluated because replicate batches and independent repeated measurements were not available. Therefore, the observed grain-size differences are interpreted as composition-dependent trends under the present experimental conditions rather than statistically verified differences. The observed variation may reflect changes in grain-growth behavior during sintering after nominal Ni incorporation and may influence grain-boundary-related interfacial polarization [23,24]. However, this interpretation remains tentative because the grain-boundary contribution was not independently separated in the present measurements.

### 3.3. X-Ray Photoelectron Spectroscopy Analysis

Considering the differences in ionic radius, electronic configuration and intrinsic magnetic moment between Ni and Fe ions, nominal Ni incorporation may modify the local structural environment and grain-growth behavior of Ba-Co U-type hexaferrite ceramics. Previous studies have demonstrated that cation substitution can regulate grain growth, grain-boundary characteristics and magnetic domain structures in hexaferrites, thereby affecting magnetic interactions and electromagnetic responses [25,26]. Therefore, XPS was employed to confirm the presence of Ni species and investigate their chemical states in the Ba-Co U-type hexaferrite ceramics.

**Figure 3:**
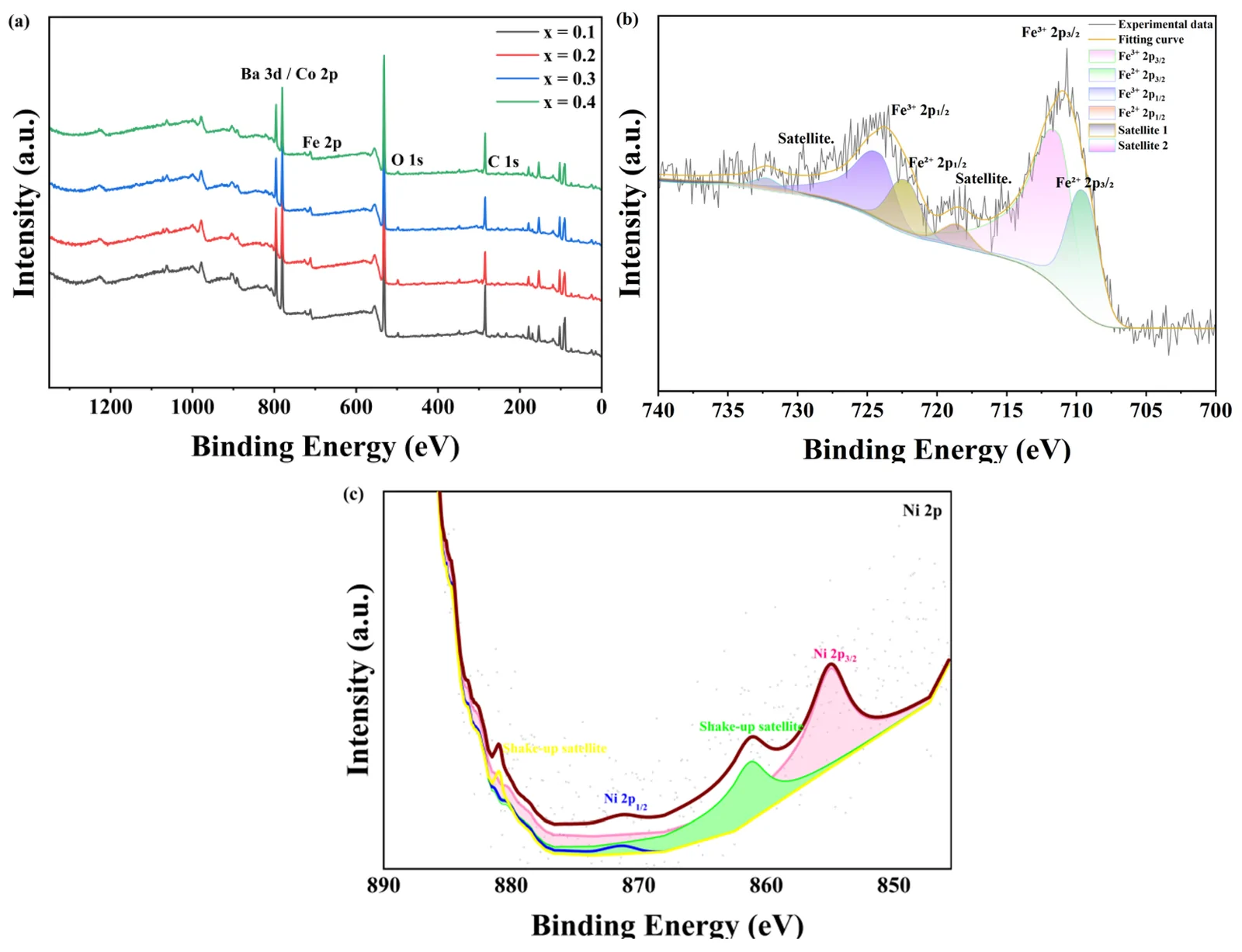
XPS spectra of Ba_4_Co_2_Fe_36−x_Ni_x_O_60_ ceramics: (**a**) survey spectra (x = 0.1–0.4); (**b**) Fe 2p spectrum of the x = 0.4 sample; and (**c**) high-resolution Ni 2p fitting spectrum of the x = 0.4 sample.

Figure 3a presents the XPS survey spectra of Ba_4_Co_2_Fe_36−x_Ni_x_O_60_ ceramics with nominal Ni contents of x = 0.1, 0.2, 0.3, and 0.4. The characteristic signals corresponding to Ba, Fe, O and Ni elements are detected, confirming the presence of Ni species in the ceramic system. The high-resolution Fe 2p spectrum of the x = 0.4 sample is shown in Figure 3b, exhibiting characteristic multiplet splitting and satellite features.

Figure 3c presents the high-resolution Ni 2p spectrum of the x = 0.4 sample. The dominant Ni 2p_3/2_ component appears at approximately 855.0 eV, accompanied by a visible shake-up satellite near 861.3 eV. These characteristic features are consistent with previously reported Ni^2+^ species in nickel-containing ferrites and Ni-substituted barium hexaferrite systems. In particular, Ni commonly exhibits a Ni 2p_3/2_ peak in the range of approximately 854.8–856.5 eV with satellite structures caused by final-state effects [27,28,29]. These spectral features are compatible with the presence of Ni^2+^ species. However, the weak Ni signal and the limitations of the component analysis prevent a reliable determination of the Ni oxidation-state distribution.

### 3.4. Magnetic Properties

**Figure 4:**
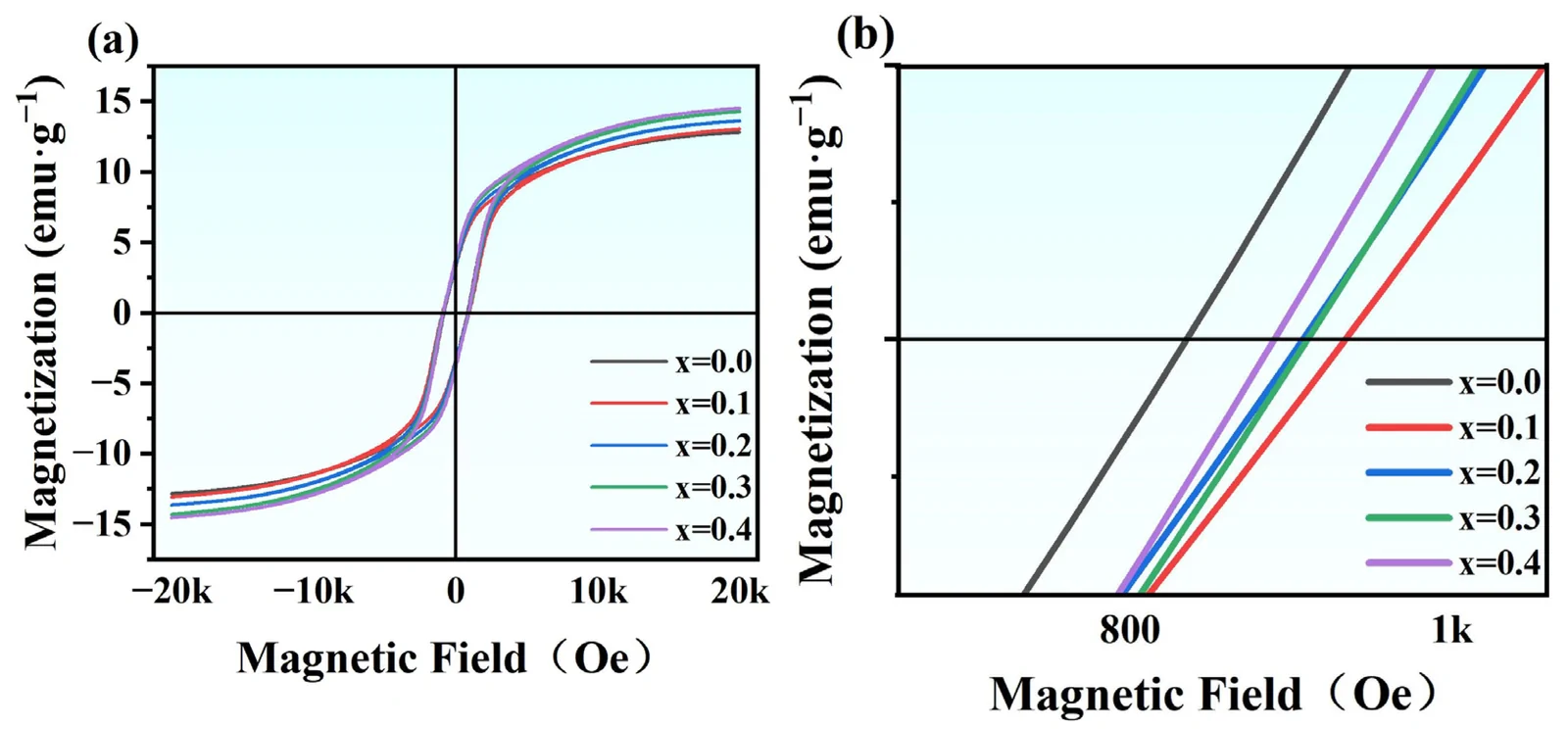
(**a**) Magnetic hysteresis loops and (**b**) enlarged view of the low-field region for coercivity analysis in Ba_4_Co_2_Fe_36−x_Ni_x_O_60_ ceramics.

Since the hysteresis loops were not fully saturated at the maximum applied field, M_s_ was approximated from the magnetization at the maximum field. Generally, although the magnetic moment of Ni^2+^ is lower than that of Fe^3+^, the saturation magnetization variation after Ni incorporation depends not only on ionic magnetic moments but also on the complex magnetic interactions in hexaferrites. The change in magnetic parameters may be related to modified magnetic interactions, microstructural evolution and domain-wall pinning after nominal Ni incorporation. Figure 4a shows the M-H hysteresis loops of the Ba_4_Co_2_Fe_36−x_Ni_x_O_60_ ceramics with different nominal Ni contents, and Figure 4b presents the enlarged low-field region. At the same time, Table 2 summarizes the core magnetic performance parameters (including saturation magnetization (M_s_), remanent magnetization (M_r_) and coercivity (H_c_)) of each sample.

M_s_ increases from 12.8 emu·g^−1^ for x = 0.0 to 14.5 emu·g^−1^ for x = 0.4, indicating that nominal Ni incorporation modifies the magnetic response of the U-type ferrite. Although the magnetic moment of Ni^2+^ is lower than that of Fe^3+^, the magnetic properties of U-type hexaferrites are governed not only by individual ionic moments but also by magnetic sublattice configuration, superexchange interactions, and microstructural factors. Previous studies on substituted U-type hexaferrites have also shown that cation substitution can affect structural and magnetic features [30]. Therefore, the increase in M_s_ is tentatively attributed to the combined effects of modified magnetic interactions, spin configurations, and microstructural evolution after nominal Ni incorporation. Specifically, Ni^2+^ may occupy multiple crystallographic sites (e.g., octahedral, tetrahedral, or trigonal-bipyramidal) in the U-type lattice, leading to complex changes in magnetic sublattice interactions rather than a single dominant mechanism. However, because direct site-occupancy evidence is unavailable, the exact contribution of Ni at specific crystallographic sites to the observed M_s_ variation cannot be determined in this work.

Figure 4b shows the enlarged low-field region of the hysteresis loops; it can be seen that the coercivity (H_c_) of the sample increases first and then decreases with the increase in Ni^2+^ substitution amount. The H_c_ of the Ba_4_Co_2_Fe_36_O_60_ sample (x = 0.0) is 835 Oe and reaches a maximum value of 934 Oe at x = 0.1. With further increasing Ni content, H_c_ generally decreases to 889 Oe at x = 0.4, despite a slight fluctuation at x = 0.3. The non-monotonic variation of H_c_ suggests that multiple factors, including magnetocrystalline anisotropy and microstructural evolution, may contribute to the observed coercivity changes. Previous studies have shown that grain-size variation can affect the coercivity of hexaferrites. In the present samples, the relatively small variation in grain size may contribute to changes in domain-wall pinning behavior, while the slight grain-size increase at higher Ni content may influence the contribution of grain-boundary-related pinning. In hexagonal ferrites, coercivity is strongly influenced by magnetocrystalline anisotropy, which originates from the anisotropic contribution of magnetic ions in the crystal lattice. Ni incorporation may modify the local magnetic environment and exchange interactions; however, the exact influence depends on the crystallographic distribution of Ni, which cannot be determined from the present results. Therefore, the variation in magnetic parameters provides a basis for understanding the microwave attenuation behavior of the samples.

### 3.5. Electromagnetic Parameters

In order to reveal the effect of Ni content on the microwave absorption properties of Ba_4_Co_2_Fe_36−x_Ni_x_O_60_ (x = 0.0, 0.1, 0.2, 0.3, 0.4) hexaferrites, the complex permittivity (*ε*ᵣ = *ε*′ − j*ε*″) and complex permeability (*μ*ᵣ = *μ*′ − j*μ*″) in the 2–18 GHz band were systematically analyzed. The real part (*ε*′, *μ*′) characterizes the storage capacity of electromagnetic energy, and the imaginary part (*ε*″, *μ*″) characterizes the loss capacity.

**Figure 5:**
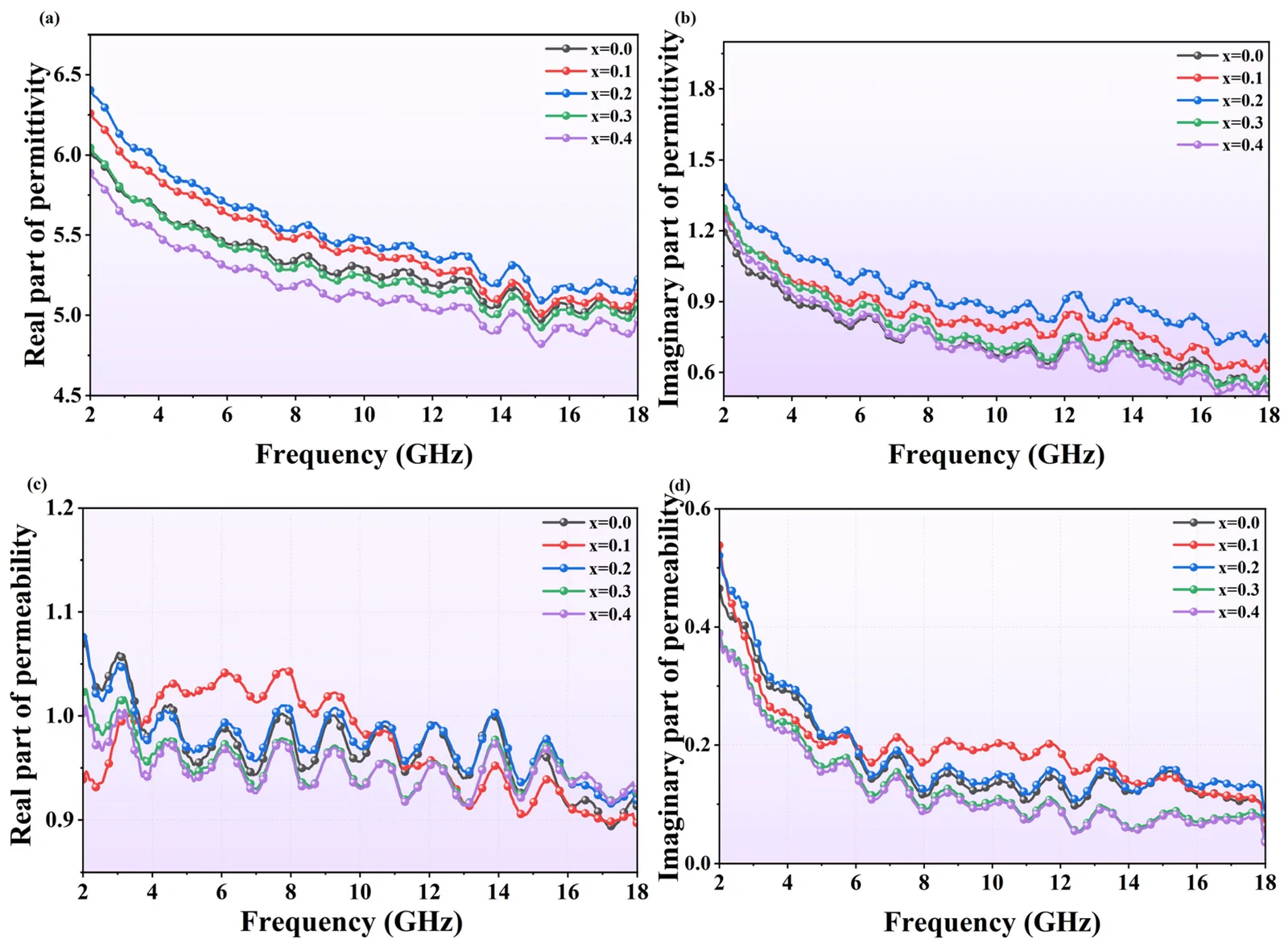
Frequency-dependent complex permittivity and permeability of Ba_4_Co_2_Fe_36−x_Ni_x_O_60_ ceramics with different Ni contents: (**a**) real part of complex permittivity, *ε*′; (**b**) imaginary part of complex permittivity, *ε*″; (**c**) real part of complex permeability, *μ*′; (**d**) imaginary part of complex permeability, *μ*″.

Figure 5a,b show the frequency dependence of *ε*′ and *ε*″ for the Ba_4_Co_2_Fe_36−x_Ni_x_O_60_ ceramics. Between 2 and 18 GHz, *ε*′ generally decreases with increasing frequency, while *ε*″ exhibits a decreasing tendency accompanied by several relaxation-related fluctuations. At 2 GHz (low frequency), *ε*′ of the x = 0.0 sample is 6.08. When x = 0.2, it increases to the maximum 6.40; when x = 0.4, it decreases to 5.88. The same rule is shown at 18 GHz: x = 0.0 is 5.10, x = 0.2 is 5.22, x = 0.4 is 4.96. The imaginary part of the dielectric constant at low frequency 2 GHz, x = 0.0 is 1.16, x = 0.2 increases to 1.38, and x = 0.4 decreases to 1.22.

The *ε*′ and *ε*″ values of the x = 0.2 sample are higher than those of the x = 0.0 and x = 0.4 samples at 2 GHz, indicating a composition-dependent dielectric response. The SEM results also reveal changes in grain-size distributions with nominal Ni content, which may modify the grain-boundary environment and contribute to interfacial polarization. Dipolar polarization may also contribute to the dielectric response. However, the present measurements cannot quantify the contributions of individual polarization processes or establish a specific charge-compensation or defect-formation mechanism. Therefore, the enhanced dielectric response at moderate nominal Ni content may be related to changes in the local polarization environment and microstructure.

The *ε*″ curve shows multiple wave-like fluctuations and several distinct dielectric relaxation peaks across the whole frequency band. The strongest relaxation peaks appear near 6–8 GHz and 12–14 GHz. According to the Debye relaxation theory, the real and imaginary parts of the dielectric constant can be expressed as [31]:

(1) $$\varepsilon^{\prime }\;=\;+\frac{\varepsilon_{s}\;-\;\varepsilon_{\infty }}{1\;+\;{\left(2\pi f\tau \right)}^{2}}$$

(2) $$\varepsilon^{\prime \prime }=\frac{\left(\varepsilon_{s}\;-\;\varepsilon_{\infty }\right)\;2\pi f\tau }{1+{\;\left(2\pi f\tau \right)}^{2}}$$

where *ε*_s_ is the static dielectric constant, *ε*_∞_ is the high-frequency limit dielectric constant, f is the applied electric field frequency, and *τ* is the dielectric relaxation time. The multiple relaxation features suggest the coexistence of different dielectric relaxation processes. The dielectric response becomes more pronounced at moderate nominal Ni contents, particularly at x = 0.2, whereas further increasing x weakens the response. This non-monotonic evolution may be associated with changes in local polarization environments and microstructural heterogeneity. Possible contributions include dipolar polarization and interfacial polarization, although their individual contributions cannot be quantitatively separated from the present measurements.

Figure 5c,d show the frequency dependence of *μ*′ and *μ*″ for the samples. As shown in Figure 5c, *μ*′ fluctuates around 1.0 over the investigated frequency range. The samples exhibit different frequency-dependent magnetic responses, with the *μ*′ values showing slight variations among different Ni contents. Generally, *μ*′ decreases gradually at higher frequencies, accompanied by several fluctuations related to magnetic relaxation processes. The variation of *μ*′ indicates that nominal Ni incorporation modifies the dynamic magnetic response of the U-type hexaferrite ceramics. Figure 5d shows that all samples maintain relatively high *μ*″ values in the low-frequency range of 2–8 GHz, indicating strong magnetic loss in this region. At 2 GHz, the *μ*″ of the x = 0.0 sample is 0.46; when x = 0.1, the maximum value is 0.53; at x = 0.4, it decreases to 0.38. As the frequency increases, *μ*″ generally decreases and remains at a relatively low value in the high-frequency region. At higher nominal Ni content (x = 0.4), the reduced *μ*″ response indicates weaker magnetic loss within the investigated frequency range. This behavior suggests that the composition-dependent evolution of the dynamic magnetic response differs from that of the static saturation magnetization. Changes in magnetic interactions and relaxation behavior may contribute to this difference; however, the individual contributions cannot be quantitatively distinguished from the present measurements.

The relatively high *μ*″ and tan*δ_μ_* values in the low-frequency region suggest that magnetic loss plays an important role in microwave attenuation. In U-type hexaferrites, magnetic loss is generally considered to originate from processes such as natural resonance and domain-wall relaxation, which are related to magnetic anisotropy, domain-wall mobility, and exchange interactions among magnetic ions. The natural resonance frequency can be qualitatively described by [32]:

(3) $$f_{r}\;=\;\frac{\gamma }{2\pi }H_{a}$$

where f_r_ is the natural resonance frequency, *γ* is the gyromagnetic ratio, and Ha is the magnetocrystalline anisotropy field. This relation indicates that the resonance behavior is closely related to the anisotropy field. Therefore, the variation in permeability after nominal Ni incorporation may be related to changes in magnetic anisotropy, magnetic interactions and microstructure. However, because H_a_ was not directly measured in this work, the formula is used only for qualitative discussion. Moreover, microwave absorption is not determined by magnetic loss alone; impedance matching between the absorber and free space is also essential.

**Figure 6:**
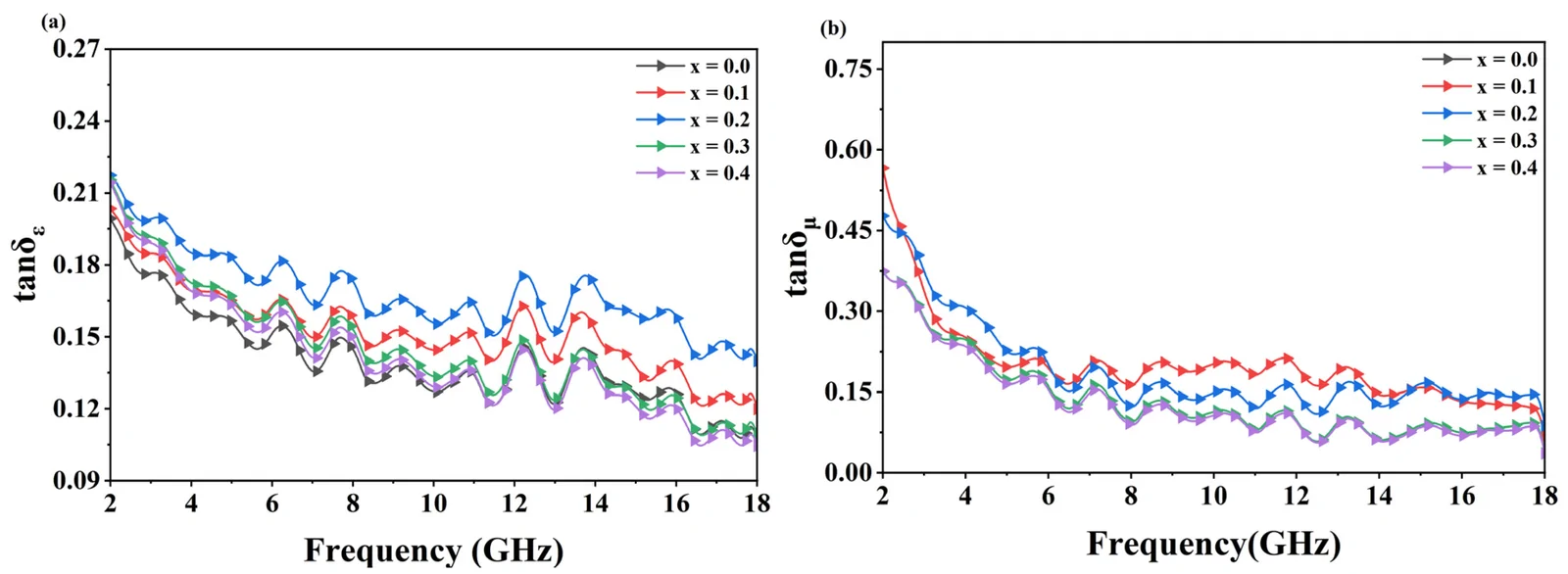
Frequency-dependent dielectric and magnetic loss tangents of Ba_4_Co_2_Fe_36−x_Ni_x_O_60_ ceramics: (**a**) dielectric loss tangent tanδ_ε_; (**b**) magnetic loss tangent tanδ*_μ_*.

Appropriate electromagnetic parameters are essential for achieving both sufficient attenuation capability and favorable impedance matching [33,34]. In this work, nominal Ni incorporation regulates the dielectric and magnetic responses of the samples. The x = 0.2 sample exhibits enhanced dielectric loss and broad −5 dB attenuation bandwidth, whereas the x = 0.4 sample achieves the strongest reflection loss. Therefore, the optimal composition depends on whether attenuation bandwidth or absorption intensity is prioritized. To further explore how Ni incorporation affects energy dissipation, the dielectric loss tangent (Figure 6a) and magnetic loss tangent (Figure 6b) were analyzed over 2–18 GHz. Because replicate batches and repeated electromagnetic measurements were not available, the present composition-dependent trends should be interpreted as experimental observations rather than statistically verified effects.

Figure 6a shows that tanδ_ε_ fluctuates across the whole frequency range. These loss peaks may originate from dipolar polarization relaxation and interfacial polarization at grain boundaries [35]. The coupling between dielectric loss and magnetic loss is also important for microwave attenuation in ferrite absorbers [36]. With increasing nominal Ni content, the positions of the dielectric loss peaks shift, indicating that nominal Ni incorporation affects the polarization relaxation behavior of the system.

Figure 6b shows that the magnetic loss tangent remains relatively high in the low-frequency range of 2–8 GHz and then gradually decreases with increasing frequency, accompanied by several resonance-related fluctuations. This behavior can be associated with resonance-related magnetic relaxation processes in hexagonal ferrites [37]. At high frequencies, magnetic loss becomes smaller, and dissipation is mainly concentrated in the low-to-mid frequency range. Taking 2 GHz as an example, the magnetic loss tangent of the sample with x = 0.1 reaches 0.56, while the dielectric loss tangent is only 0.20. As x increases to 0.4, tanδ*_μ_* decreases to 0.38. Over most of the investigated frequency range, tanδ*_μ_* is higher than tanδ_ε_, suggesting that magnetic loss makes an important contribution to electromagnetic energy dissipation, particularly in the low-to-mid-frequency region. Nevertheless, the overall microwave absorption performance is jointly governed by dielectric loss, magnetic loss, attenuation capability and impedance matching.

**Figure 7:**
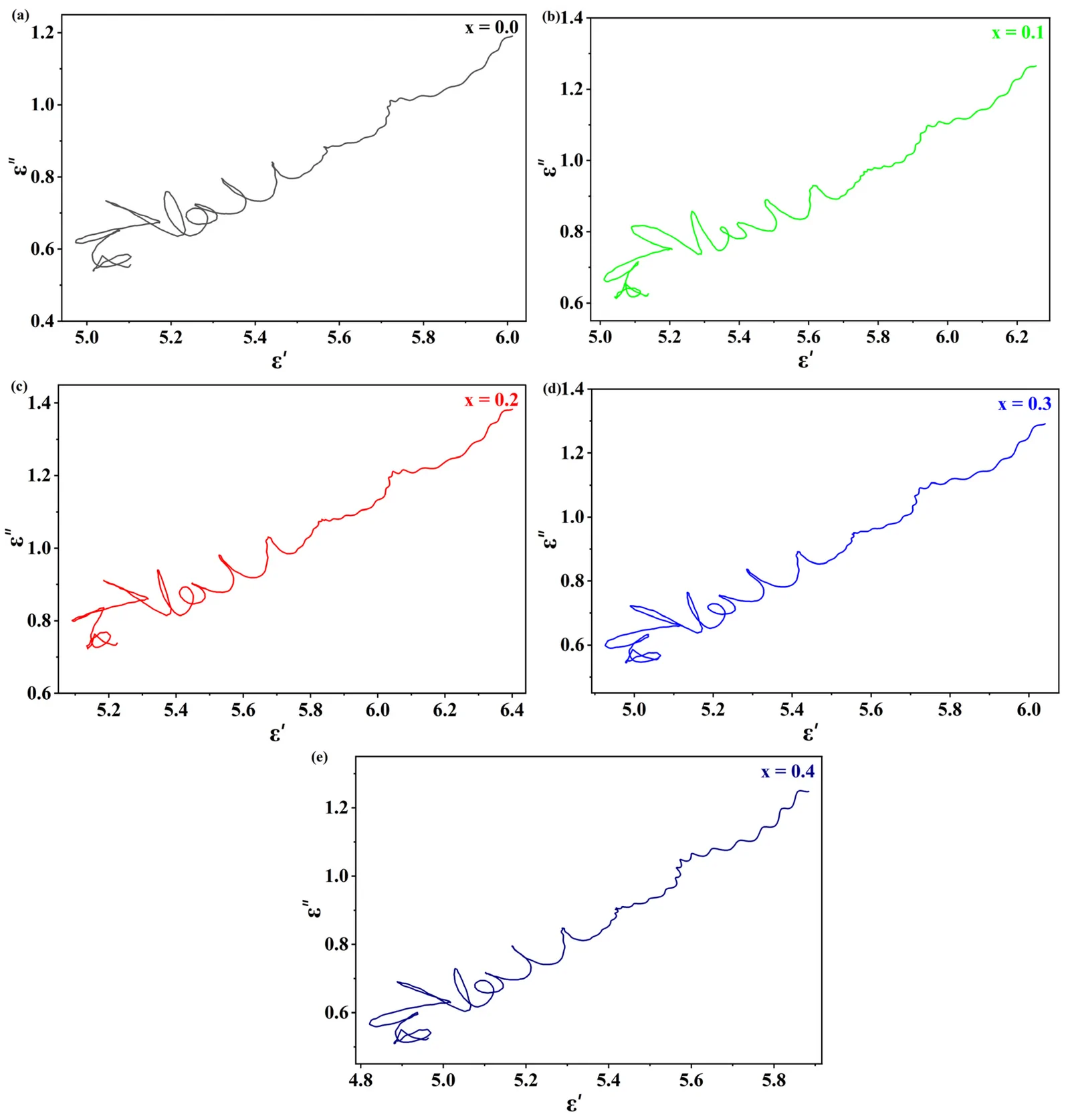
Cole–Cole plots of Ba_4_Co_2_Fe_36−x_Ni_x_O_60_ ceramics with different Ni contents: (**a**) x = 0.0; (**b**) x = 0.1; (**c**) x = 0.2; (**d**) x = 0.3; (**e**) x = 0.4.

To further clarify the dielectric relaxation mechanism, Cole–Cole plots were constructed based on the measured *ε*′ and *ε*″ values, as shown in Figure 7. The deviation from ideal semicircular behavior indicates the existence of multiple dielectric relaxation processes rather than a single Debye relaxation process. Moreover, the depressed semicircular arcs observed in the Cole–Cole plots suggest the contribution of multiple relaxation centers, which may be associated with dipolar and interfacial polarization in heterogeneous regions. A specific defect-related mechanism cannot be confirmed from the present measurements alone. The variation of Cole–Cole curves with Ni incorporation indicates that the dielectric relaxation behavior is affected by changes in polarization environments and heterogeneous microstructures. Combined with the chemical-state information from XPS analysis and the heterogeneous grain morphology observed by SEM, these relaxation behaviors may be associated with heterogeneous regions and grain boundaries, which contribute to interfacial polarization processes.

More specifically, the x = 0.2 sample exhibits the largest *ε*″ range and more pronounced loop-like features in the Cole–Cole plot, indicating relatively strong and heterogeneous dielectric relaxation. The x = 0.4 sample shows a lower *ε*′ range and a reduced maximum *ε*″, suggesting a weakened polarization response at higher nominal Ni content. The x = 0.1 and x = 0.3 samples display intermediate relaxation characteristics. These differences indicate that nominal Ni incorporation changes both the relaxation strength and the heterogeneity of the polarization environment. Because no equivalent-circuit fitting or independent relaxation spectroscopy was performed, the individual features cannot be assigned to unique microscopic defect species.

The evolution of the Cole–Cole plots is consistent with changes in the dielectric relaxation behavior and polarization heterogeneity after nominal Ni incorporation. However, the Cole–Cole plots alone cannot identify the specific defect species or quantitatively determine the number of independent relaxation centers.

In summary, nominal Ni incorporation tunes the electromagnetic parameters and loss behavior of Ba-Co U-type hexaferrite ceramics, leading to a balance between impedance matching and attenuation capability.

### 3.6. Microwave Absorption Properties

In this work, the effective absorption bandwidth was evaluated using the criterion of RL ≤ −5 dB and RL ≤ −10 dB. The RL ≤ −5 dB corresponds to a reflected-power ratio below approximately 31.6%, indicating that at least 68.4% of the incident electromagnetic power is absorbed. The input impedance, reflection loss, normalized input-impedance magnitude, and attenuation constant were calculated as follows [38]:

(4) $$Z_{in}=Z_{0}\sqrt{\frac{\mu_{r}}{\varepsilon_{r}}}tanh\left[j\frac{2\pi fd}{c}\sqrt{\mu_{r}\varepsilon_{r}}\right]$$

(5) $$\mathrm{RL}\left(\mathrm{dB}\right)=20\log_{10}\frac{\left|Z_{\mathrm{in}}-Z_{0}\right|}{\left|Z_{\mathrm{in}}+Z_{0}\right|}$$

(6) $$C_{0}=\left|\frac{Z_{in}}{Z_{0}}\right|$$

(7) $$\alpha =\frac{\sqrt{2}\pi f}{c}\sqrt{(\mu^{“}\varepsilon^{“}-\mu^{\prime }\varepsilon^{\prime })+\sqrt{(\mu^{“}\varepsilon^{“}-{\mu^{\prime }\varepsilon^{\prime })}^{2}+({\mu^{\prime }\varepsilon^{“}+\mu^{“}\varepsilon^{\prime })}^{2}}}$$

where *C*_0_ is the normalized input-impedance magnitude, and a value approaching unity indicates favorable impedance-magnitude matching between the absorber and free space. Here, *Z*_in_ is the input impedance of the absorber, *Z*_0_ is the impedance of free space, *μ*_r_ and *ε*_r_ are the complex permeability and permittivity, respectively, d is the absorber thickness, f is the frequency, and c is the speed of light in a vacuum. *α* is the attenuation constant, while *ε*’ and *ε*″ denote the real and imaginary parts of the complex permittivity, and *μ*′ and *μ*″ denote the real and imaginary parts of the complex permeability, respectively.

**Figure 8:**
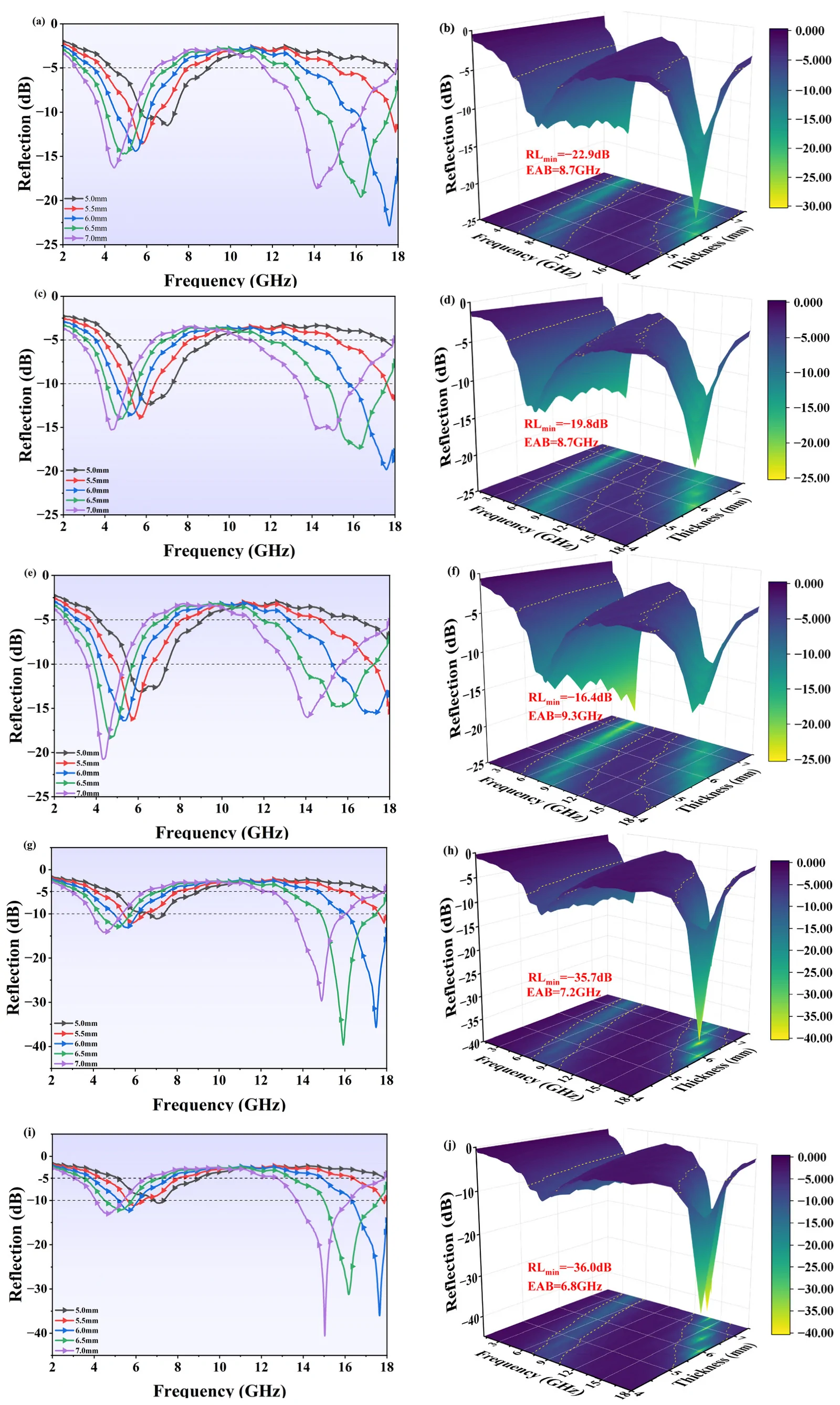
Reflection loss curves and corresponding three-dimensional reflection loss maps of Ba_4_Co_2_Fe_36−x_Ni_x_O_60_ ceramics with different Ni contents at various absorber thicknesses: (**a**,**b**) x = 0.0; (**c**,**d**) x = 0.1; (**e**,**f**) x = 0.2; (**g**,**h**) x = 0.3; (**i**,**j**) x = 0.4.

Figure 8 shows the two-dimensional reflection loss curves and corresponding three-dimensional reflection loss maps of the samples at various absorber thicknesses. At an absorber thickness of 6.0 mm, the RL_min_ values of the samples with nominal Ni contents of x = 0.0, 0.1, 0.2, 0.3 and 0.4 were −22.9, −19.8, −16.4, −35.7 and −36.0 dB, respectively. The corresponding −10 dB absorbing bandwidths were 3.6, 3.7, 4.6, 3.1 and 2.6 GHz, respectively. The x = 0.2 sample exhibited the broadest effective absorption bandwidth, whereas the x = 0.4 sample achieved the RL_min_ = −36.0 dB. These results suggest that the optimal composition depends on whether attenuation bandwidth or absorption intensity is prioritized. The enhanced microwave attenuation behavior may be associated with the regulated electromagnetic parameters, favorable impedance matching and the synergistic contribution of dielectric and magnetic losses. Similar phenomena have been reported in substituted ferrite, where compositional regulation optimizes the balance between attenuation capability and impedance matching [39,40,41]. Although the x = 0.4 sample does not exhibit the highest dielectric or magnetic loss over the whole frequency range, its RL_min_ of −36.0 dB indicates a favorable balance between attenuation capability and impedance matching. The normalized input impedance magnitude (|Z_in_/Z_0_|) and attenuation constant (α) were further analyzed to evaluate the contribution of impedance matching and attenuation. Table 3 summarizes the microwave absorption performance of our optimized U-type hexaferrite sample and representative reported U, Y, Z, and M-type hexaferrite absorbers.

**Figure 9:**
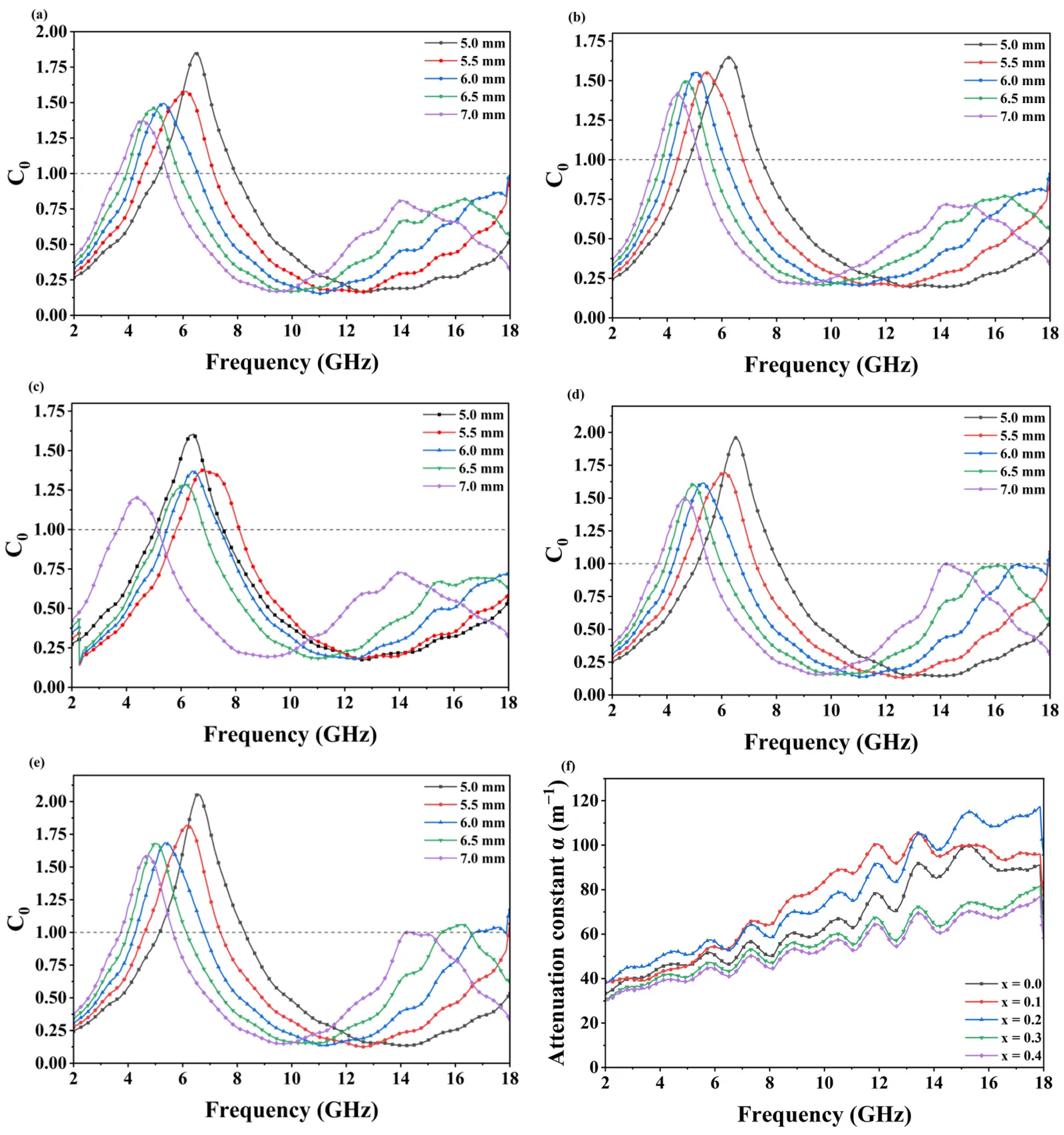
Frequency dependence of the normalized input impedance *C*_0_ = |Z_in_/Z_0_|of Ba_4_Co_2_Fe_36−x_Ni_x_O_60_ ceramics at absorber thicknesses of 5.0–7.0 mm: (**a**) x = 0.0; (**b**) x = 0.1; (**c**) x = 0.2; (**d**) x = 0.3; (**e**) x = 0.4. (**f**) Frequency dependence of the attenuation constant α for samples with different Ni contents. The horizontal dashed lines in (**a**–**e**) represent *C*_0_ = 1.

To further evaluate the impedance-matching behavior, the frequency-dependent *C*_0_ values at different absorber thicknesses are presented in Figure 9a–e. All samples exhibit pronounced thickness-dependent matching characteristics, and the main matching peaks gradually shift toward lower frequencies with increasing absorber thickness. Ni incorporation changes the frequency positions and widths of the near-unity matching regions, indicating that the balance between complex permittivity and permeability is effectively tuned. Therefore, the reflection-loss performance originates from the combined effects of impedance matching and intrinsic electromagnetic attenuation rather than from dielectric or magnetic loss alone.

Figure 9f further compares the attenuation constants of the samples. The x = 0.2 sample generally exhibits a higher α value over a broad mid-to-high-frequency range, indicating stronger intrinsic electromagnetic attenuation. In contrast, although the x = 0.4 sample shows a relatively lower attenuation constant, it achieves the strongest reflection loss. This result further confirms that strong microwave absorption depends on the balance between attenuation capability and impedance matching.

## 4. Conclusions

In this study, Ni-substituted Ba_4_Co_2_Fe_36−x_Ni_x_O_60_ (x = 0.0–0.4) U-type hexaferrite ceramics were prepared by a conventional solid-state reaction method. XRD results suggest that the samples are mainly composed of a U-type hexaferrite-related phase, while trace secondary phases below the detection limit of laboratory XRD cannot be completely excluded. SEM observations reveal faceted and polyhedral grains with polygonal and triangular outlines and non-monotonic grain-size evolution with Ni incorporation. The saturation magnetization increases from 12.8 to 14.5 emu·g**^−1^**, while the coercivity first increases and then decreases, suggesting that nominal Ni incorporation modifies the magnetic response of the ferrite ceramics. Electromagnetic measurements show that Ni incorporation tunes the complex permittivity and permeability. Among the samples with different Ni contents, the x = 0.2 sample exhibits an absorbing width of 9.3 GHz (RL < −5 dB) in a thickness of 6.0 mm, whereas the x = 0.4 sample achieves the RL_min_ = −36.0 dB. The improved microwave attenuation behavior is governed by the combined effects of dielectric loss, magnetic loss, attenuation capability and impedance matching. These results demonstrate that nominal Ni incorporation is an effective compositional-regulation strategy for balancing attenuation bandwidth and absorption intensity in Ba-Co U-type hexaferrite ceramics, while the impedance-matching analysis further demonstrates that Ni incorporation modifies the thickness-dependent matching behavior over 2–18 GHz.

## Figures and Tables

**Table 1:** Statistical analysis of grain size for Ba_4_Co_2_Fe_36−x_Ni_x_O_60_ samples.

Sample	Average Grain Size (μm)	Standard Deviation (μm)
x = 0.0	0.659	0.324
x = 0.1	0.577	0.294
x = 0.2	0.606	0.231
x = 0.3	0.570	0.290
x = 0.4	0.647	0.314

**Table 2:** Magnetic parameters of Ba_4_Co_2_Fe_36−x_Ni_x_O_60_ (x ≤ 0.4) ceramics as a function of Ni content.

Sample	M_s_ (emu·g^−1^)	M_r_ (emu·g^−1^)	H_c_ (Oe)
x = 0.0	12.8	3.3	835
x = 0.1	13.1	3.3	934
x = 0.2	13.6	3.4	907
x = 0.3	14.3	3.7	910
x = 0.4	14.5	3.8	889

**Table 3:** Comparison of microwave absorption performance and representative U/Y/M/Z-type hexaferrite absorbers.

Sample	Thickness (mm)	RL_min_ (dB)	EAB (GHz)	Ref.
Ba_4_Co_2_Fe_36−x_Ni_x_O_60_	6.0	−36	6.8	This work
Ba_4_Ni_x_Co_2−x_Fe_36_O_60_	2.5	−24.4	4.8	[37]
Dy^3+^–Cu^2+^-doped Co_2_Y hexaferrite	3.5	−18	1.5	[42]
Co–Ca co-substituted Ba-M hexaferrite	2.0	−59.5	3.31	[43]
Ba_2_._7_La_0_._3_Co_2_Fe_24_O_41_	3.5	−47.3	4.32	[44]

## Data Availability

The data presented in this study are available on request from the corresponding author.
